# Plasmonic Light Emission by Inelastic Charge Transport in Ultrathin Zinc Oxide/Metal Heterostructures

**DOI:** 10.1021/acs.nanolett.4c06099

**Published:** 2025-02-04

**Authors:** Henrik Wiedenhaupt, Fabian Schulz, Luis E. Parra López, Adnan Hammud, Youngwook Park, Akitoshi Shiotari, Takashi Kumagai, Martin Wolf, Melanie Müller

**Affiliations:** † Department of Physical Chemistry, Fritz-Haber Institute of the Max-Planck Society, Faradayweg 4-6, 14195 Berlin, Germany; ‡ Department of Inorganic Chemistry, Fritz-Haber Institute of the Max-Planck Society, Faradayweg 4-6, 14195 Berlin, Germany; § Institute for Molecular Science, 38 NishigoNaka, Myodaiji, Okazaki 444-8585, Japan; ∥ The Graduate University for Advanced Studies, SOKENDAI, Hayama, Kanagawa 240-0193, Japan

**Keywords:** Plasmonic nanocavities, scanning tunneling microscopy, STM luminescence, inelastic electron transport, ultrathin oxides, metal/oxide interfaces

## Abstract

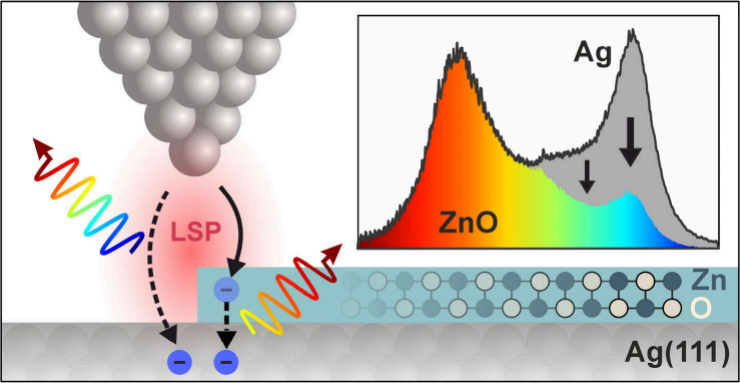

Controlling light emission from plasmonic nanojunctions is crucial for developing tunable nanoscale light sources and integrated photonic applications. It requires precise engineering of plasmonic nanocavity electrodes and a detailed understanding of electrically driven light emission. Using scanning tunneling microscopy-induced luminescence (STML), we studied plasmonic light emission from ultrathin ZnO/Ag(111) inside a silver nanocavity. At positive bias, plasmonic luminescence, caused by radiative decay of localized surface plasmons (LSP), is spectrally low-pass filtered by the ZnO layers. The emission of photon energies above the conduction band edge energy (***E***_**CB**_) of ZnO is suppressed, while the spectral distribution below ***E***_**CB**_ resembles the LSP resonance on Ag(111). This spectral filtering is absent at negative bias and depends on the local electronic structure, as confirmed by spatial STML mapping. Our findings demonstrate that the ZnO conduction band serves as the initial state for plasmonic luminescence driven by inelastic electron transport across the ZnO/Ag(111) interface.

Plasmonic nanocavities can confine and enhance electromagnetic fields at the nanometer and subnanometer scale, leading to strongly enhanced and highly localized light-matter interaction. This is frequently realized in low-temperature scanning tunneling microscopy (STM), where stable formation of plasmonic nanocavities and picocavities^1,2^ between the tip and substrate enables optical imaging and spectroscopy^3−8^ as well as strong enhancement and control of electrically driven light emission at atomic scales.^9−17^ As a specific manifestation of electroluminescence, STM-induced luminescence (STML), in particular, is a powerful tool for characterizing light emission from plasmonic nanocavities through radiative decay of localized surface plasmons (LSP) excited by inelastic electron tunneling (IET), and for investigating charge transport and radiative recombination pathways in optically active nanostructures.

The precise control of electroluminescence from plasmonic nanocavities is important for advancing our understanding of nanoscale light-matter interaction and for developing sensitive and tunable nanooptical light sources. In general, the LSP and its emission characteristics can be controlled by the geometry and dielectric environment of the nanocavity^18^ down to the atomic scale.^17,19^ In addition, electrically driven plasmonic luminescence can be modulated by the local density of electronic states (LDOS) involved in IET. On metal surfaces, spatial variations in plasmonic luminescence have been reported due to oscillations in the LDOS of a two-dimensional electron gas^20^ and in one-dimensional atomic chains.^21^ Molecular monolayers have been shown to modify the plasmonic luminescence^9,22^ or induce spectral shifts of the LSP resonance.^22,23^ Molecular states can further act as spatially and energetically confined nanogates for plasmon excitation.^24^ Moreover, a shifted quantum cutoff has been observed for electronically decoupled ultrathin oxide or semiconductor films.^25−27^ While for metal surfaces, the maximum emitted photon energy is determined by the STM bias, the quantum cutoff can be shifted to higher bias in the presence of an electronic gap, inside which the final state LDOS vanishes. A shifted quantum cutoff has been also observed for inelastic tunneling into quantum-well states,^28,29^ defect states,^26^ field emission resonances^30^ (FERs) or molecular states.^31^ Opposed to these final state effects, it has been reported that occupied molecular states^31^ or FERs^32,33^ can alter the initial states in IET. While IET from molecular initial states leads to a modified quantum cutoff,^31^ resonant tunneling through FERs can enhance plasmonic luminescence by increasing the inelastic tunneling rate.^32^ In all cases, it is the IET through the vacuum barrier that is ultimately responsible for LSP excitation and plasmonic light emission.

Here, we report a fundamentally different mechanism of plasmonic light emission mediated by inelastic electron transport at the interface between ultrathin ZnO and Ag(111) within a plasmonic nanocavity. In contrast to LSP excitation by IET across the tip–sample gap, plasmonic light emission is driven by charge injection into the conduction band (CB) of ZnO and subsequent radiative decay into bulk Ag states. This results in a bias-independent high-energy cutoff, which is determined by the energy difference *E*_CB_ between the ZnO conduction band edge (CBE) and the Fermi level of the Ag(111) substrate. Our results show that the CBE of ultrathin ZnO serves as a new initial state for plasmonic luminescence, where the emission spectrum is low-pass filtered compared to light emission from the bare plasmonic nanocavity.

Ultrathin ZnO on Ag(111) has emerged as an interesting material platform for the atomic-scale investigation of light-matter interaction in plasmonic tunnel junctions.^34−37^ As ultrathin layers, ZnO forms a two-dimensional hexagonal lattice with a layer-thickness dependent electronic structure^38^ that varies at the nanoscale.^35^ In addition, an interface state (IS) is formed at the ZnO-Ag(111) interface, derived from the Shockley surface state on Ag(111). Previous work has shown that the coupling of the ZnO–CBE to the IS plays an important role in the plasmon-enhanced optical excitation of ZnO/Ag(111). In particular, the IS-CBE resonance leads to enhancement in tip-enhanced Raman spectroscopy (TERS)^34,36,37^ and enables detection of coherent phonons by resonant photoassisted ultrafast STM.^35^ However, a detailed understanding of the coupling of the ZnO electronic states with the Ag(111) substrate and the plasmonic nanocavity is still lacking and requires further investigations.

Figure 1a shows a sketch of the STM junction formed by a silver tip and the ZnO/Ag(111) sample. Applying a positive or negative sample bias *V*_b_ allows electrons to tunnel from the tip to the sample or vice versa. At sufficiently high bias, the tunneling electrons have enough energy to induce plasmonic luminescence via IET from an occupied initial state into empty sample or tip states, as illustrated in Figure 1b for a bare Ag–Ag junction. Figure 1c shows an STM image of typical ZnO islands on Ag(111) with areas of 2 ML-ZnO (dark-gray; cyan star) and 3 ML-ZnO (light gray; dark blue star). Due to a lattice mismatch between the ZnO and the Ag(111) surface, a hexagonal moiré pattern with ∼2.3 nm periodicity is formed.^38−40^

**Figure 1:**
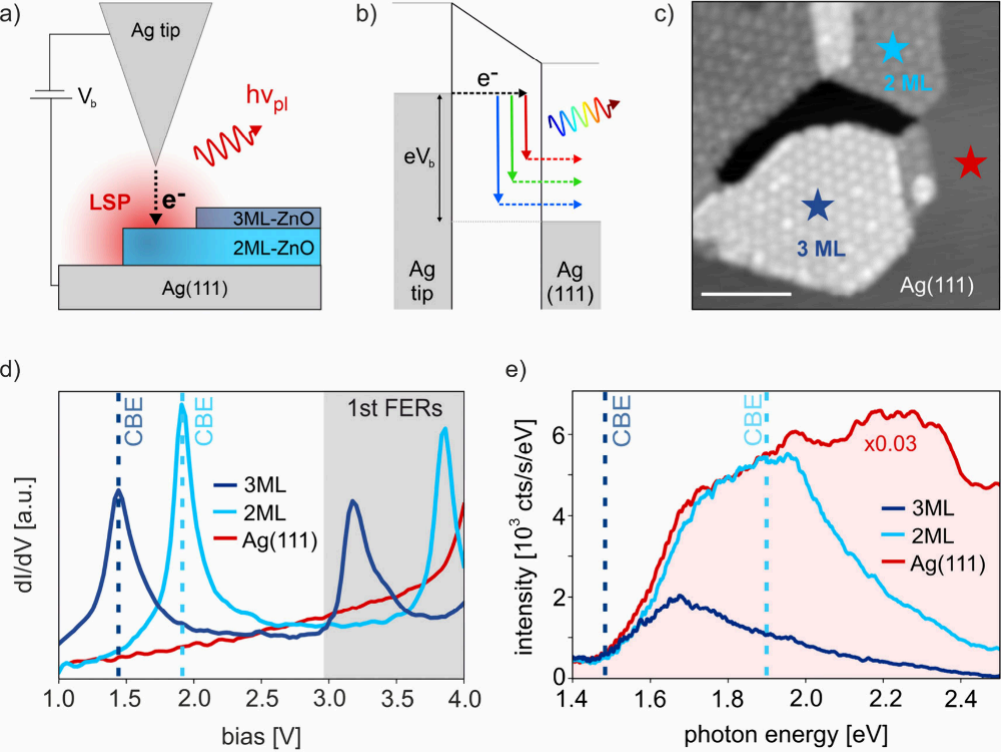
STML of ultrathin ZnO/Ag(111). a) Concept of tunneling-induced STM luminescence. The Ag tip and Ag(111) substrate form a plasmonic nanocavity, which can contain 2 and 3 ML films of ZnO epitaxially grown on Ag(111). b) Schematic energy diagram of the Ag–Ag junction and broadband light emission driven by IET. c) Topographic STM image of ZnO islands on Ag(111) (*I*_t_ = 10 pA, *V*_b_ = 1 V, scale bar 10 nm). Dark blue, light blue and red stars mark the position of the spectra shown in d) and e). d) Constant current STS spectra recorded on 2 ML-ZnO, 3 ML-ZnO and Ag(111). The peaks at 1.45 and 1.90 eV can be assigned to the ZnO CB edges. e) STML spectra recorded on Ag(111) (red, 3 V), 2 ML-ZnO (light blue, 3 V) and 3 ML-ZnO (dark blue, 2.5 V). The reference spectrum on Ag(111) is scaled for better comparison. Vertical dashed lines indicate the CBE onset of 2 ML-ZnO and 3 ML-ZnO.

To characterize the layer-dependent electronic structure and CBE position of the ultrathin ZnO films, we performed scanning tunneling spectroscopy (STS) as shown in Figure 1d. STS spectra are recorded in constant current mode so that the CBE appears as a peak in the differential conductance (dI/dV) as the tip retracts with increasing *V*_b_. Defining the center of the STS peak as the CBE position, its energy *E*_CB_ shifts from 1.90 eV on 2 ML-ZnO to 1.45 eV on 3 ML-ZnO.^34,38^ The additional peaks at 3.2 V on 3 ML-ZnO and 3.8 V on 2 ML-ZnO correspond to the first FER states, respectively.^38^ The IS (not shown here) is located at −0.2 V.^34,38^

The LSP resonance is characterized by recording STML spectra on Ag(111) at 3 V bias. The red spectrum in Figure 1e shows the broad emission due to the LSP, whereby the exact spectral shape depends on the geometry and size of the tip apex.^18,41^ Next, we measured STML on ultrathin ZnO. The light blue curve shows the STML spectrum on 2 ML-ZnO (cyan star in Figure 1c) recorded at the same bias and set point current of 3 V and 8 nA, respectively. In addition to a suppression of the total STML intensity by a factor of ∼30, we find a broad luminescence spectrum, indicating a plasmonic origin. However, its shape is significantly modified compared to the spectrum recorded on Ag(111). For a better comparison of the spectral distributions, the spectrum recorded on Ag(111) has been rescaled by a factor of 0.03. While we observe almost identical spectral shapes at photon energies up to ∼2 eV, the shape of the STML spectrum on 2 ML-ZnO starts to deviate significantly from the bare Ag spectrum at photon energies ≳2 eV, where the intensity decreases continuously with increasing photon energy. The observed suppression of photon energies > *E*_CB_ is independent of the spectral shape of the LSP. Figure S1 in the Supporting Information (SI) shows a similar behavior for an LSP including two radiative modes. Interestingly, we find a similar behavior for STML recorded on 3 ML-ZnO (dark blue curve), where compared to 2 ML-ZnO the onset of suppression occurs at lower photon energies and the intensity continuously decreases for *E*_ph_ ≳ 1.6 eV. This layer-dependent onset of suppression of high photon energies strongly indicates a contribution of ZnO electronic states to light emission from the biased nanocavity.

To better understand the origin of the observed spectral changes, we recorded STML spectra on ZnO for varying positive sample bias. Figures 2a and 2b show STML intensity maps and selected line spectra (bottom panel) measured on 2 ML-ZnO and 3 ML-ZnO, respectively. For better comparison of spectral shapes, each STML spectrum (horizontal line) is normalized to its maximum at a given bias (note that the integrated and peak STML intensities depend on the STM bias, as discussed below). The dashed yellow line shows the quantum cutoff, as determined by the STM bias. Interestingly, we find a constant spectral shape independent of STM bias for both 2 ML-ZnO and 3 ML-ZnO. Note that at low sample bias close to the red tail of the LSP, the normalized spectra appear shifted towards low photon energies due to the quantum cutoff. Similar to Figure 1e, the STML intensity is suppressed at high photon energies, and the spectrum is low-pass filtered compared to luminescence from Ag(111) (top panels).

**Figure 2:**
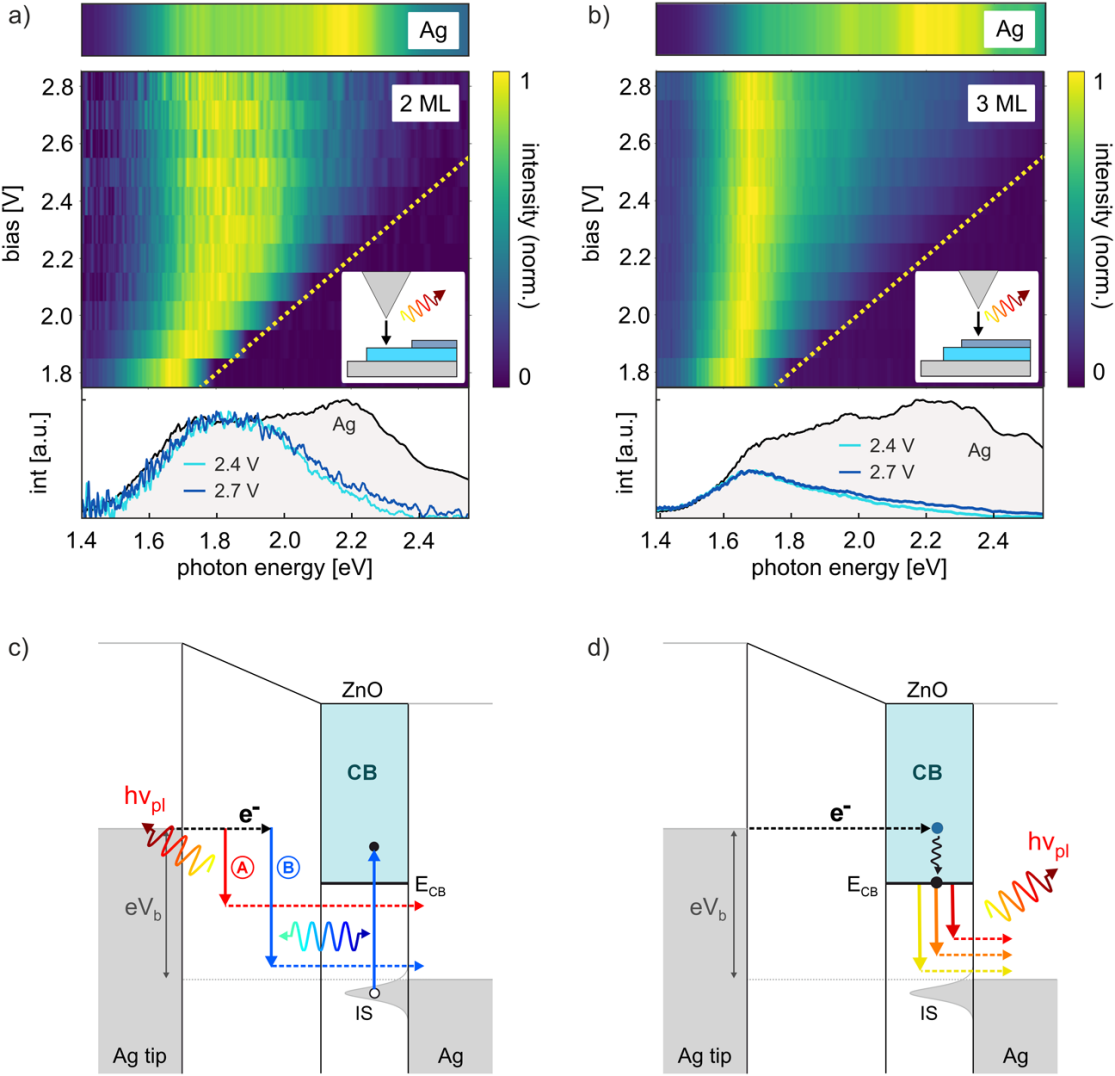
STML at positive sample bias. Bias dependence of STML spectra on (a) 2 ML-ZnO and (b) 3 ML-ZnO when electrons tunnel from tip to sample (see insets) at positive sample bias. Each spectrum (horizontal line) is normalized to its maximum. The yellow dashed lines show the quantum cutoff. The top panels show the STML reference spectra on bare Ag(111) recorded at 2.8 V. The bottom panels show line spectra (horizontal cuts) at two different bias voltages and for the bare Ag(111) reference spectrum. c) Sketch of energy transfer between the LSP excited via inelastic tunneling and optical transitions in the ZnO/Ag(111). Low energy photons cannot excite transitions in ZnO/Ag(111) and are thus emitted (channel A), while for high energy photons energy transfer leads to damping (channel B). d) Sketch of plasmonic luminescence driven by charge injection into the CB of ZnO via elastic tunneling. After relaxation to the CBE, the tunneled electron can radiatively decay through LSP excitation, resulting in broadband emission with a maximum photon energy defined by *E*_ph_ ≤ *E*_CB_.

Importantly, while the spectral filtering does not depend on the magnitude of the bias voltage, it does depend on the ZnO layer thickness and correlates with *E*_CB_. For 2 ML-ZnO, the luminescence decreases significantly for photon energies *E*_ph_ ≳ 2 eV, which is close to *E*_CB,2 ML_ = 1.9 eV as defined by the STS peak position in Figure 1d. On 3 ML-ZnO, the luminescence decreases for photon energies *E*_ph_ ≳ 1.6 eV, which is slightly higher than the CBE position of 3 ML-ZnO at *E*_CB,2 ML_ = 1.45 eV. Note, however, that assigning an onset for spectral low-pass filtering is difficult for 3 ML-ZnO because the red tail of the LSP energetically overlaps with the CBE position, so that the STML spectra are limited by the spectral enhancement function of the LSP at low photon energies. Finally, we can conclude that for both 2 ML-ZnO and 3 ML-ZnO, the emission of photons with energy larger than *E*_CB_ is strongly suppressed even at large sample bias *eV*_b_ > *E*_CB_.

The above results imply that ultrathin ZnO acts as a spectral low-pass filter for the plasmonic luminescence emitted from the silver nanocavity, where the wavelength-dependent decrease of luminescence intensity depends on the ZnO layer thickness. Two possible mechanisms explain the observed behavior. In single-molecule STML studies, resonant energy transfer between the LSP and molecular excitons has been observed.^12,42^ In this process, the LSP is excited by IET between the tip and the metal substrate and subsequently couples to the molecules via a dipole–dipole interaction. A similar scenario could occur in ZnO as sketched in Figure 2c. IET into empty states in the Ag(111) substrate can excite the LSP, which can then interact with the ZnO layer. In particular, absorption of photons with energy *E*_ph_ > *E*_CB_ could promote electron transfer from occupied states in bulk Ag or from the IS into the CB of ZnO. If the excited electrons decay nonradiatively, e.g. by scattering into Ag bulk states or by transport out of the plasmonic near-field region, this would create a nonradiative loss channel for photons with energies high enough to excite electron–hole pairs in ZnO/Ag(111). In contrast, photons with energies *E*_ph_ < *E*_CB_ cannot excite ZnO/Ag(111) and are emitted via radiative decay of the LSP. This process would depend on the optical transitions in ZnO/Ag(111), but would be independent of the polarity of the applied bias.

Another possible mechanism is charge injection into the CB as sketched in Figure 2d. At high positive bias *eV*_b_ > *E*_CB_, electrons from the tip can elastically tunnel into the CB of ZnO, where they can relax to the band edge by electron–electron and electron–phonon scattering. After relaxation, electrons residing at the CBE could lose energy by radiative decay into unoccupied states above *E*_F_ through LSP excitation. Assuming that the electronic final states are the same as in IET from the tip to Ag(111) (Figure 1b), the STML spectrum recorded on ZnO will have the same spectral shape as on bare Ag(111), but only up to *E*_ph,max_ = *E*_CB_ as determined by the CBE energy. In this scenario, the CBE serves as a new energetically lower initial state for plasmonic luminescence generated via inelastic electron transport from ZnO to Ag(111). As a result, the plasmonic STML spectrum will exhibit a bias-independent cutoff at *E*_CB_, above which photon emission will be suppressed.

The two mechanisms can be distinguished by measuring STML at negative sample bias on 2 ML-ZnO and 3 ML-ZnO as shown in Figure 3a and 3b. In contrast to positive bias (Figure 2), no wavelength-selective suppression is observed, and the spectra on ZnO closely resemble the full plasmonic spectrum measured on Ag(111) (upper panels in Figure 3a and 3b). This is further evident from the line spectra shown in Figure 3c. The spectra on 2 ML-ZnO and 3 ML-ZnO are almost identical and, apart from a slight red shift, are also almost identical to the Ag(111) reference spectrum. A red-shifted plasmonic luminescence has been observed previously for molecular layers on metal surfaces,^23,31,43^ and was explained by the dielectric polarizability of the oxide layer.^44^

**Figure 3:**
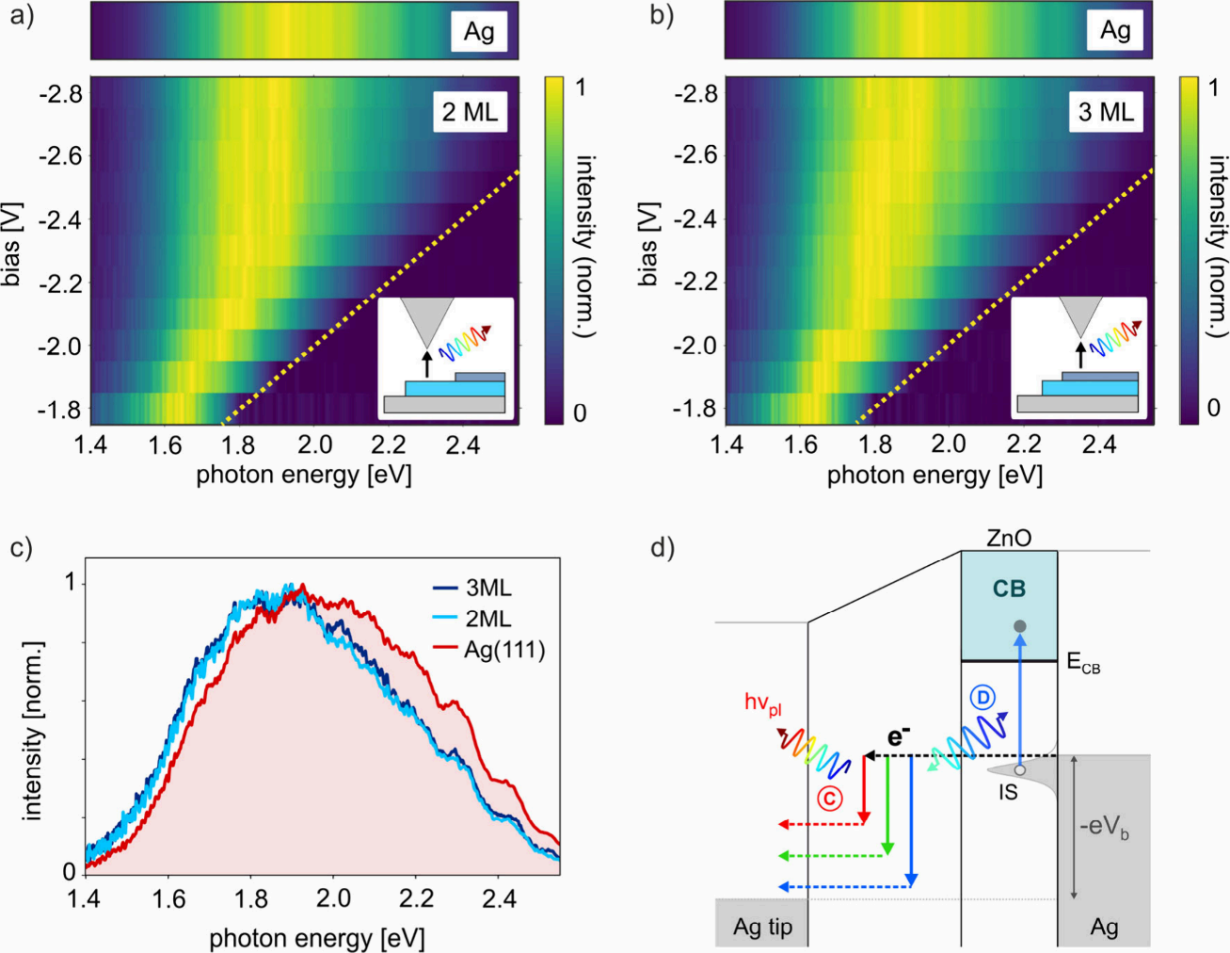
STML at negative sample bias. Bias dependence of normalized STML spectra on (a) 2 ML-ZnO and (b) 3 ML-ZnO when electrons tunnel from sample to tip (see insets) at negative sample bias. As in Figure 2, each spectrum (horizontal line) is normalized to its maximum, and the yellow dashed lines and the top panels show the quantum cutoff and STML spectra on Ag(111) for *V*_b_ = 3 V, respectively. Note that the tip condition is not the same as in Figure 1 and 2. (c) Comparison of STML line spectra recorded on Ag(111) (red, 3 V), 2 ML-ZnO (light blue, −2.8 V), and 3 ML-ZnO (dark blue, −2.8 V). d) Sketch of broadband plasmonic luminescence induced by IET from Ag(111) to the Ag tip at negative sample bias on ZnO (channel C). No CB states of ZnO are involved in tunneling, such that charge injection into ZnO cannot influence the emitted spectra. The nearly identical spectra in (a-c) proof that energy transfer between the LSP and the ZnO/Ag(111) via dipole–dipole coupling (channel D) is negligible.

At negative bias, electrons tunnel from the sample to the tip, and charge injection into the ZnO–CB does not occur. Considering the absence of valence band states in ultrathin ZnO/Ag(111) in a bias window down to −4 V,^38^ the only mechanism for light emission is IET from the Ag substrate or the IS into empty tip states. Similar to Figure 2c and as sketched in Figure 3d, the excited LSP could interact with ZnO/Ag(111) via dipole–dipole interaction. However, the coupling of the LSP to optical transitions in the ZnO/Ag(111) should be independent of the direction of IET, and one would expect the same luminescence spectra for both polarities of the STM bias. The fact that we do not observe any spectral filtering at negative sample bias, but measure the full plasmonic spectrum, rules out such a dipole–dipole interaction. Instead, our results show that charge injection into the CB and subsequent inelastic electron transport from the CBE to the Ag(111) substrate is an efficient channel for plasmonic light emission from ZnO/Ag(111).

In addition to radiative decay as sketched in Figure 2d, nonradiative losses might occur due to scattering of electrons into Ag(111) bulk states. This has been observed on thin Cu_2_O films, where a decrease of STML intensity at high bias voltages above the CBE of Cu_2_O was explained by nonradiative losses into the Au substrate.^33^ A more detailed understanding of such losses can be obtained from the bias dependence of the STML intensity. Figure 4a shows the same bias-dependent STML data as in Figure 2a, but plotted in units of cts/s/eV without normalization to their respective maxima. This allows a quantitative comparison of different bias voltages. We find that the peak STML intensity exhibits a pronounced maximum when tunneling at *E*_CB_ (cyan dashed line), and decreases significantly for *eV*_b_>*E*_CB_ when tunneling into higher-lying CB states.

**Figure 4:**
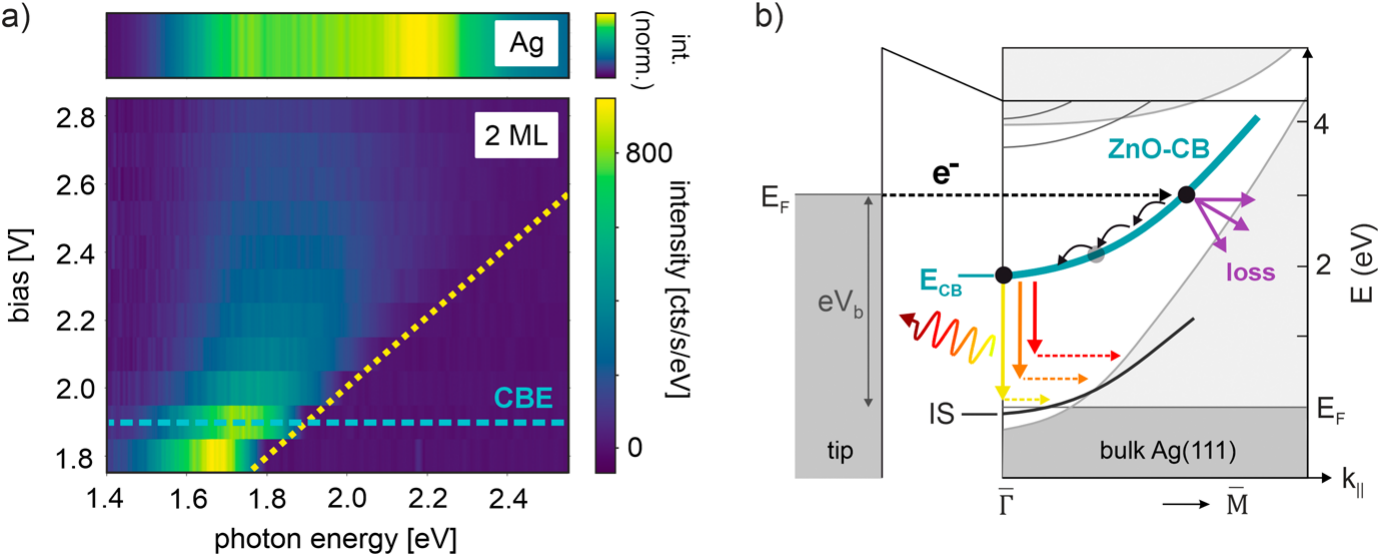
Bias-dependent STML intensity. a) Bias dependence of raw STML spectra on 2 ML-ZnO at positive sample bias when tunneling from tip to sample. The peak STML intensity decreases when tunneling at high energy above *E*_CB_, indicating a bias-dependent contribution from nonradiative losses. The yellow dashed line marks the quantum cutoff. The blue horizontal line marks the CBE position. The top panel shows the STML reference spectrum on bare Ag(111) recorded at 2.8 V. b) Schematic band diagram of ZnO/Ag(111). Dark (light) gray regions mark occupied (empty) states in the Ag(111) substrate. No electronic states are available in Ag(111) in the white region. Electrons injected into higher-lying ZnO–CB states can decay nonradiatively into the Ag(111) bulk via elastic and inelastic scattering, or relax down to the CBE, from where they can decay radiatively into empty Ag(111) states. No empty states are available at the Gamma point, but the highly localized LSP might provide the momentum mismatch that is required for the radiative decay into states away from the Gamma point as sketched.

At high positive bias, electrons are injected into CB states with high energy and momentum, as sketched in Figure 4b, with the Ag(111) bulk projected band structure adopted from literature,^45,46^ and the dispersion of ZnO–CB and IS bands estimated from previous calculations.^34^ The larger energy and momentum of electrons injected at high bias allows them to scatter more easily into nearby bulk Ag electronic states, creating a nonradiative loss channel (purple arrows). This could explain the continuously decreasing STML intensity for increasing bias voltages above the CBE. However, a decreasing luminescence yield at high bias could also be caused by a decreasing plasmonic enhancement due to a bias-dependent increase in the gap size. The effect of gap size changes on the STML intensity are discussed in the SI (sections 2 and 3). Although they are likely to contribute to the observed decrease of STML intensity, the very strong decrease we observe at higher bias voltages suggests that nonradiative losses also play a role.

The CBE of ZnO at the Gamma point is located in the middle of the bulk band gap of Ag(111). Therefore, electrons scattering into Ag bulk states from the CBE require a larger momentum transfer compared with high-energy CB states. Together with the decreased phase space for e-e-scattering, this decreases the probability for nonradiative losses at the CBE. On the other hand, radiative decay from the CBE into empty Ag states or the unoccupied part of the IS via LSP excitation also requires a small momentum transfer. We thus speculate that the highly localized near-field of the LSP facilitates the necessary momentum transfer for radiative decay and plasmonic luminescence from ZnO/Ag(111). Although a contribution of the IS could be expected considering the larger wave function overlap of the ZnO–CB and the IS, a clear identification of the final states involved in STML from ZnO/Ag(111) is difficult. Overall, we conclude that the observed bias-dependent decrease in STML intensity can be rationalized by a higher probability of radiative decay of electrons residing near the CBE, while electrons at higher energies undergo nonradiative scattering into Ag(111) as competing loss channel. Specifically, we speculate that the favorable alignment of the ZnO–CB within the Ag(111) bulk band gap reduces nonradiative scattering losses, extending the charge state lifetime in the CB and enabling plasmonic luminescence from the CBE.

We further note that the residual photon emission at *E*_ph_ > *E*_CB_ could originate from IET between the tip and Ag(111) without a contribution of the ZnO, or from hot luminescence, i.e. radiative decay of electrons from higher-lying CB states above the CBE. If IET through the vacuum gap would dominantly contribute, normalization of the STML spectra to the inelastic tunneling rates^13^ would yield the same high-energy tail for ZnO/Ag(111) and Ag(111), which is not the case as we show in Figure S6 in the SI. We therefore conclude that the high-energy tail originates from hot luminescence, enhanced by the strong coupling of the LSP to optical transitions in the ZnO–CB/Ag(111) heterostructure, in agreement with previous observations.^34,35^

Finally, we measure the spatial dependence of STML spectra in the proximity of an edge and across a ZnO flake of varying thickness. Figure 5a shows a topographic STM image of a ZnO flake that contains areas of 2 ML-ZnO (yellow regions) and 3 ML-ZnO (blue-colored regions). Figure 5b shows STML spectra at *V*_b_ = 2.5 V at positions 1 to 8 as marked in Figure 5a. We observe constant STML on Ag(111) in close proximity of the ZnO, with no dependence on tip position (positions 1 and 2). As we move the tip on ZnO, electrons can tunnel into the ZnO–CB and the plasmonic luminescence exhibits the low-pass filtered spectrum with suppression of photon energies *E*_ph_ > *E*_CB_. Scanning the tip across a region of adjacent 3 ML-ZnO and 2 ML-ZnO shows spectral changes similar to those observed in Figures 1 and 2 (note that the data have been recorded with different tip conditions). As evident from the spatial profiles of the topography (Figure 5c) and STML intensity at three different photon energies (Figure 5d), the spectra change abruptly within the used step size (more STML mapping at positive and also negative sample biases is available in sections 3 and 4 of the SI). Furthermore, STML mapping on a spatially inhomogeneous ZnO island with a spatially undefined electronic structure confirms the need for a well-defined CBE and the local nature of the luminescence signal. This corroborates that charge injection into the ZnO–CB is responsible for the suppression of photons with *E*_ph_>*E*_CB_, and demonstrates the ability to control electrically driven plasmonic light emission on the nanoscale.

**Figure 5:**
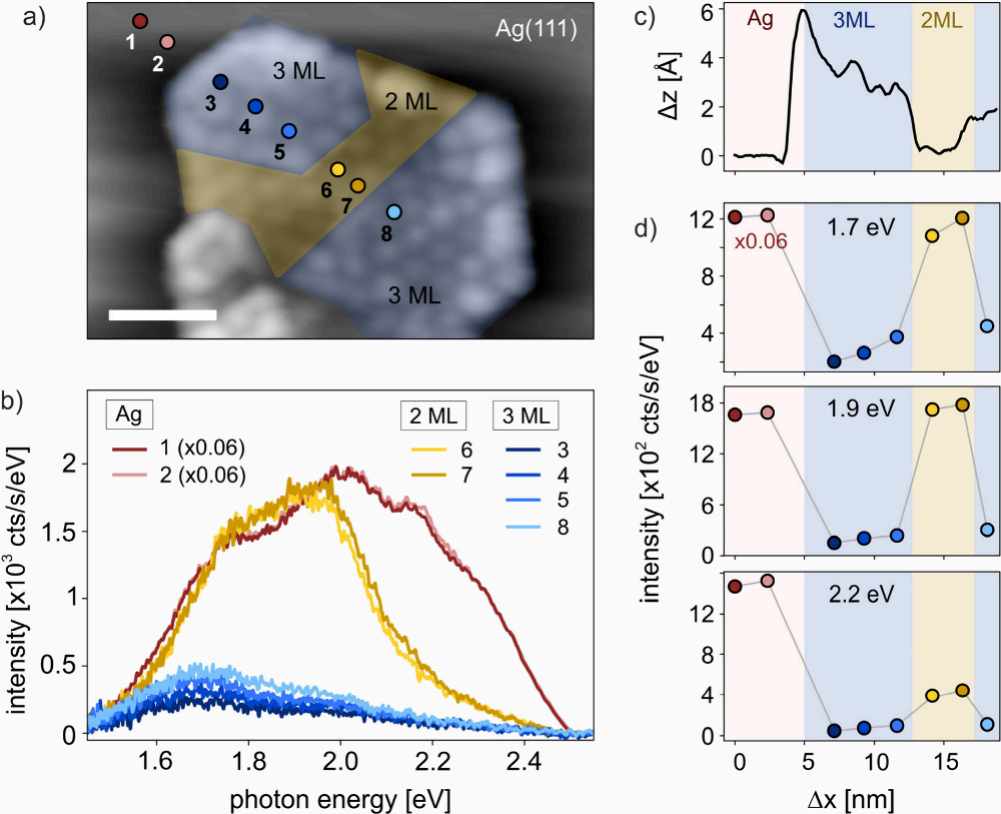
Spatial mapping of STML on ZnO/Ag(111). a) STM image of a ZnO flake of varying thickness. Yellow-shaded regions are 2 ML-ZnO and blue-shaded regions are 3 ML-ZnO. (*V*_b_ = 1 V, *I*_t_ = 10 pA, scale bar 5 nm). b) STML spectra recorded at different tip positions with the numbers as marked in a). To account for the overall decrease of STML intensity on ZnO compared to Ag(111), the spectra on Ag(111) are multiplied by 0.06 for better comparison of spectral shapes. (*V*_b_ = 2.5 V). c) Topography and d) STML intensity at photon energies *E*_ph_ = 1.7 eV, *E*_ph_ = 1.9 eV and *E*_ph_ = 2.2 eV as a function of lateral distance Δ*x*. As in b), the data points on Ag(111) are multiplied by a factor of 0.06 to account for the overall decrease of STML intensity at high positive bias (Figure 4).

In summary, we demonstrated electrically driven plasmonic luminescence from ZnO/Ag(111) heterostructures inside a biased plasmonic nanocavity. The emitted photons are low-pass filtered due to charge injection into the ZnO conduction band compared with the emission from the bare plasmonic junction. Importantly, the high-energy cutoff for photon emission can be controlled by the local electronic structure of the ZnO films, and the spectral distribution and yield of the electroluminescence can vary abruptly on the nanoscale. The plasmonic luminescence occurs via radiative decay of the LSP excited by inelastic electron transfer from ZnO–CB into Ag(111) bulk states. This mechanism should also occur in other ultrathin oxides, semiconductors, or insulators on metal substrates, but we expect its efficiency to strongly depend on the charge state lifetime in the nonmetallic layer and the enhancement of radiative decay by the plasmonic nanocavity. Our findings reveal a new mechanism for plasmonic luminescence that provides a previously unexplored route to control broadband light emission from plasmonic nanostructures at atomic scales.

## Methods

The experiments were performed at 10 K in a UHV chamber comprising a low-temperature STM (modified UNISOKU USM-1400) operated with a Nanonis SPM controller enabling STM operation at a base pressure below 4 × 10^–10^ mbar. The bias voltage was applied to the sample, and the tunneling current was collected from the tip. STS was performed in constant current mode at a tunneling set point of 100 pA and a bias modulation amplitude and frequency of 20 mV and 933 Hz, respectively.

Ag tips are electrochemically etched from polycrystalline Ag wire. The end of the tip shaft (50 μm length) is polished by focused ion beam (FIB) milling, and the tip is cleaned by Ar^+^ sputtering inside UHV after transport in air. The single-crystal Ag(111) surface was cleaned by repeated cycles of Ar^+^ sputtering and annealing up to 670 K. Ultrathin ZnO was epitaxially grown on Ag(111) using reactive deposition^47^ inside UHV at a base pressure below 5 × 10^–10^ mbar. Zn is deposited on Ag(111) at room temperature in O_2_ atmosphere (1 × 10^–5^ mbar), and the sample is then annealed at 700 K.^47^

The emitted photons are collected and collimated by a silver off-axis parabolic mirror (OAPM) with a numerical aperture of 0.6 mounted on the cold STM stage. The collected light is focused into a grating spectrometer (AndorShamrock 303i) with a water-cooled CCD attached to it outside the STM chamber. Linear movement in three dimensions and two rotational axes allows for precise alignment of the OAPM and the photon collection beam path. Typical accumulation times for recording the STML spectra are between 30 and 120 s. All STML spectra have been measured at a tunneling current of 8 nA. Before normalization, all spectra were converted from wavelengths to eV according to a Jacobian conversion.^48^

## Supporting Information Available

1. Influence of tip condition and LSP spectral shape; 2. Bias-dependence of STML intensity at positive sample bias, 3. STML mapping at negative sample bias, 4. Extended data: spatial STML mapping; 5. STML on a spatially inhomogeneous 2 ML-ZnO island, 6. Normalization of STML spectra to the inelastic tunneling rate (PDF)
