# Supplementary material for: Plasmonic Light Emission by Inelastic Charge Transport in Ultrathin Zinc Oxide/Metal Heterostructures
Source: Nano Lett. 2025 Feb 4;25(7):2870–7. doi: 10.1021/acs.nanolett.4c06099 (PMC11848997; doi:10.1021/acs.nanolett.4c06099)
Supplement: Supplementary file 1 — nl4c06099_si_001.pdf [file nl4c06099_si_001.pdf]

Supporting Information:

Plasmonic Light Emission by Inelastic Charge Transport in Ultrathin Zinc Oxide/Metal Heterostructures

Henrik Wiedenhaupt[1], Fabian Schulz[1#], Luis E. Parra López[1], Adnan Hammud[2], Youngwook Park[1], Akitoshi Shiotari[1], Takashi Kumagai[3,4], Martin Wolf[1], Melanie Müller[1]*

[1]*Department of Physical Chemistry, Fritz-Haber Institute of the Max-Planck Society, Faradayweg 4-6, 14195 Berlin, Germany.*

[2]*Department of Inorganic Chemistry, Fritz-Haber Institute of the Max-Planck Society, Faradayweg 4-6, 14195 Berlin, Germany.*

[3]*Institute for Molecular Science, 38 NishigoNaka, Myodaiji, Okazaki 444-8585, Japan*

[4]*The Graduate University for Advanced Studies, SOKENDAI, Hayama, Kanagawa, 240-0193, Japan*

*Corresponding author: m.mueller@fhi-berlin.mpg.de

[#] Present address: CIC nanoGUNE, Tolosa Hiribidea 76, 20018 San Sebastian, Spain.

Table of Content:

1. **Influence of tip condition and LSP spectral shape**
2. **Effect of z-piezo retraction on STML intensity**
3. **STML mapping at negative sample bias**
4. **Extended data: spatial STML mapping**
5. **STML on a spatially inhomogeneous 2ML-ZnO island**
6. **Normalization of STML spectra to the inelastic tunneling rate**

1. Influence of tip condition and LSP spectral shape

The low-pass filter behavior of ZnO and the suppression of luminescence at high photon energies is independent from the tip condition and the exact shape of LSP resonance. Figure S1 shows STML on 2ML-ZnO compared to that on Ag(111) for a different tip. The STML on Ag(111) reveals two pronounced plasmonic modes centered around 1.8 eV and 2.3 eV. On 2ML-ZnO, the spectral shape of the low energy LSP mode at 1.8 eV remains unchanged up to a photon energy of ~1.95 eV, which corresponds to the STS peak and the onset of the CB. For higher photon energies, the STML intensity is increasingly suppressed with increasing photon energy. Interestingly, the high-energy plasmon mode around 2.3 eV is still visible, but is significantly suppressed. This clearly shows that the luminescence on ZnO/Ag(111) is of plasmonic origin, but is strongly low-pass filtered by the ZnO layer. The remaining STML intensity with photon energies above E_{CB} can originate from IET between the tip and the Ag(111) substrate, and/or from hot luminescence, i.e. radiative decay of electrons from higher-lying CB states into the Ag(111) bulk.

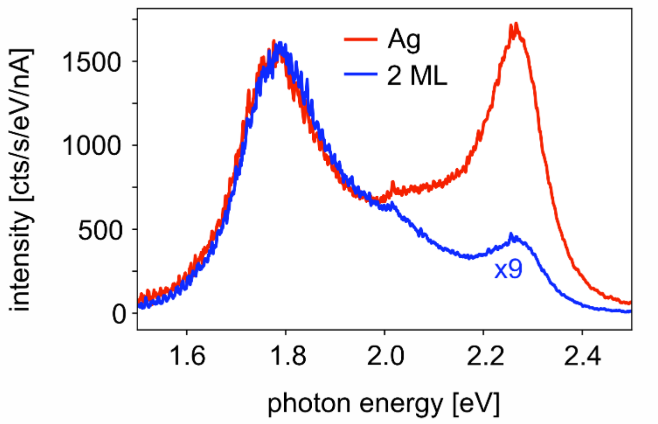

Figure S1. STML spectra taken on Ag(111) and 2ML-ZnO at 5 nA and 70 nA, respectively (V_b = 3 V). The spectrum on 2ML-ZnO is multiplied by a factor of 9 for better comparison.

2. Bias-dependence of STML intensity at positive sample bias

Besides the bias-independent high-energy emission cutoff, we find that the overall STML intensity varies strongly with the applied positive sample bias. Specifically, it exhibits a pronounced maximum when the Fermi level of the tip aligns energetically with the onset of the CB of ZnO, and decreases significantly for higher bias voltages when tunneling into higher-lying CB states (Figure 4a for 2ML-ZnO). Figure S2a shows the corresponding STML intensity at 1.7 eV (orange squares) and the integrated STML intensity (dark circles) as a function of the applied bias. The integrated STML intensity peaks around 1.8 V, above which it sharply drops

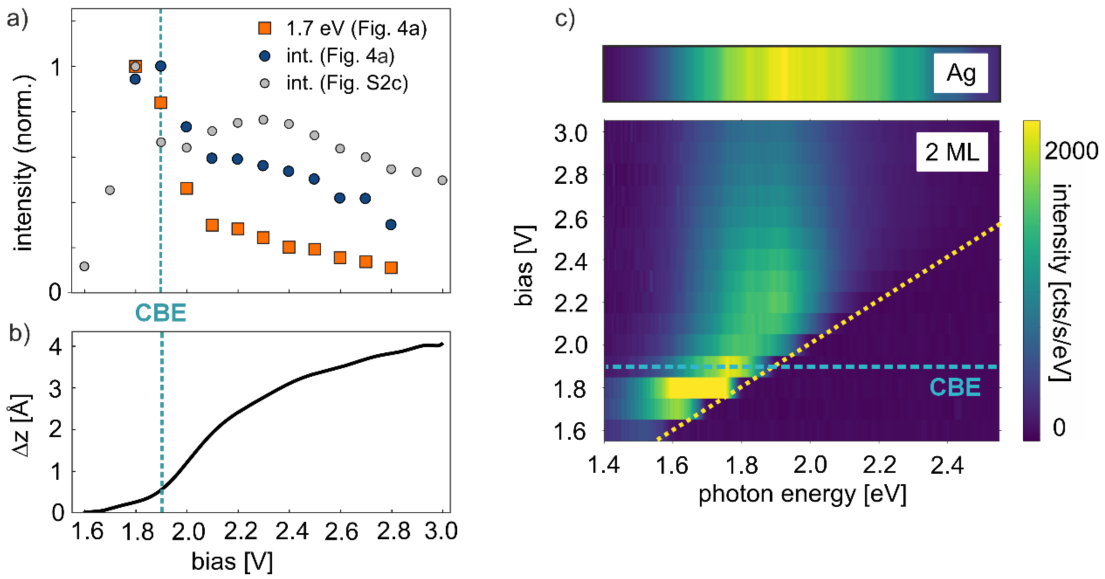

Figure S2) a) Dependence of STML intensity on sample bias for the data shown in Figure 4. Orange squares are the STML intensity at 1.7 eV photon energy, dark circles show the integrated intensity. b) Retraction of the z-piezo with increasing sample bias on 2ML-ZnO at a constant current of $I_t = 8$ nA. c) Another data set of STML spectra versus positive sample on 2ML-ZnO, recorded with a different tip condition as in Fig. 2a (Normalized LSP spectra on Ag(111) shown in the top panel).

and then keeps decreasing slowly up to the highest used bias of 2.8 V. The light grey circles show the integrated STML intensity for another data set shown in Figure S2c over a slightly larger bias range.

Two main effects can contribute to a decreasing STML intensity at higher bias voltages: non-radiative losses and a decreasing plasmonic enhancement due to retraction of the z-piezo and a corresponding increase in gap size, since the STML spectra are recorded in constant current mode. As can be seen in Figure S2b), the z-piezo retracts by ~0.5 Å when changing the bias from 1.6 V to 1.9 V, and further retracts by ~1 Å in a small bias window from 1.9 eV to 2.1 eV around the CBE, in which a new conductance channel for tunneling into the CB of ZnO opens. At higher bias, the gap size keeps slightly increasing by another ~2-3 Å up to a bias of 3 V.

The sharp drop of the STML intensity correlates with the highest slope of the tip retraction, so it can be expected that a decreasing field enhancement due to tip retraction at least partially contributes to the observed decrease of the integral STML intensity. From current-dependent STML measurements on Ag(111), we estimate that a retraction of ~1 Å (corresponding to a decrease of the current from 95 nA to 10 nA at 2 V) leads to a decrease of the integrated STML intensity of about 38 %. This is less than the almost 80% decrease of STML intensity at 1.7 eV we observe in the small bias range around the CBE, in which the tip retracts by roughly ~1 Å. We therefore conclude that a change in gap size alone cannot explain the steep decrease of STML intensity above the CBE, and that non-radiative losses, as sketched in Figure 4b, also

contribute to the strongly and continuously decreasing luminescence yield. In particular, at high bias, the z-piezo retraction becomes very small so that no significant changes to the LSP enhancement are expected, but the STML continues to decrease. Overall, the bias-dependent change of the gap size and the resulting decrease of plasmonic field enhancement renders a quantitative analysis of non-radiative losses difficult in the current experiments.

Finally, we note that an analysis of non-radiative losses in 3ML-ZnO is difficult in the present experiments because the CBE of 3ML-ZnO of 1.45 eV lies outside the plasmonic enhancement function of our Ag-nanocavities. Specifically, for a quantitative comparison of the layer-thickness dependence of non-radiative losses, we would need to measure the relative decrease of STML intensity on 3ML-ZnO for high bias far above the CBE compared to STML at bias voltages around 1.45 eV, which for 3ML-ZnO lies outside the LSP resonance of the nanocavity required to enhance the radiative decay.

3. STML mapping at negative sample bias

Figures S3b) and S3c) show STML spectra for the different tip positions when scanning from Ag(111) to 2ML-ZnO (Figure 3a) at positive and negative sample bias, respectively. At positive bias, we observe the same low-pass filter behavior and overall decrease of STML intensity on ZnO as in Figure S1 and in the main manuscript. The STML spectra in Figure S3b) are therefore normalized for better comparison of their spectral shapes. Interestingly, at negative bias, not only the spectral shape but also the STML intensity remains almost constant when the tip is moved from Ag(111) to 2ML-ZnO, and the overall decrease in STML intensity we observe at positive sample bias $> E_{\mathrm{CB}}$ does not occur.

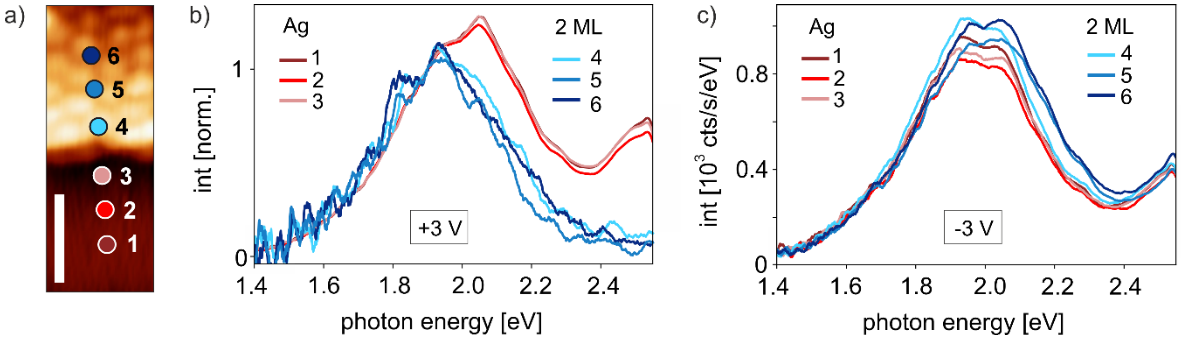

Figure S3. a) Topographic STM image of a 2ML-ZnO edge (scale bar: 4 nm). b) STML spectra recorded at positive sample bias (3 V, I_t = 8 nA) for the tip positions shown in (a). The spectra are normalized for better comparison of their spectral shape. c) STML spectra recorded at negative sample bias (-3 V, I_t = 8 nA) at the same positions. The spectra are plotted in units of cts/s/eV and show comparable intensities and spectral shapes on Ag(111) and 2ML-ZnO, respectively.

When tunneling at -3 V, the z-piezo retracts by ~4 Å when the tip is moved from Ag(111) to 2ML-ZnO. Therefore, the fact that we observe the same STML intensity on 2ML-ZnO and Ag(111) suggests that small changes in the gap distance between the tip and the Ag(111) substrate do not significantly affect the LSP enhancement, supporting that non-radiative losses dominate the decrease of STML intensity at high positive sample bias $eV_{\mathrm{b}} > E_{\mathrm{CB}}$.

4. Extended data: spatial STML mapping

Figure S4 shows another data set of position-dependent STML spectra recorded on the same ZnO island as shown in Figure 4. The results are qualitatively and quantitatively the same as in Figure 5, but the distance dependence on Ag(111) is a more extensive with one more STML spectrum at a larger lateral distance towards the ZnO island. No spatial STML dependence on Ag(111) is observed in close proximity to the ZnO edge, excluding energy transfer between the ZnO and the LSP.[1,2]

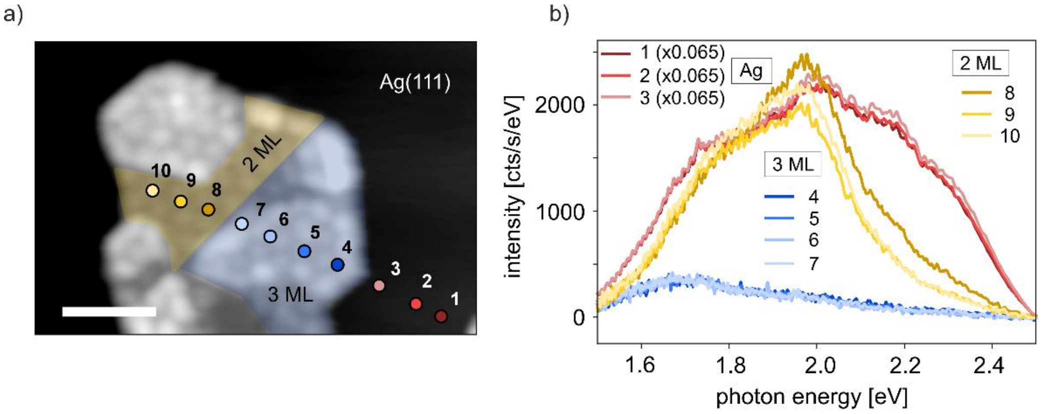

Figure S4. a) STM image of a ZnO island of varying thickness. Yellow shaded regions are 2ML-ZnO and blue shaded regions are 3ML-ZnO. (V_b=1 V, I_t=10 pA, scale bar 6 nm). b) STML recorded spectra at different tip positions with the numbers as marked in a). To account for the overall decrease of STML intensity on ZnO compared to Ag(111), the spectra on Ag(111) are multiplied by 0.065 for better comparison of spectral shapes. (V_b=2.5 V, I_t=8 nA).

5. STML on a spatially inhomogeneous 2ML-ZnO island

At liquid nitrogen temperature, it is possible to induce disorder in the ultrathin ZnO islands by applying a high bias (close to the ZnO work function) and high current for roughly one minute after positioning the tip on ZnO. This allows us to study the dependence of the plasmonic luminescence from ZnO/Ag(111) on the quality of the electronic structure of the ZnO films.

The well-ordered moiré pattern is apparent in the STM image and STS map at the CBE (Figures S5a and S5b). STS scans at different locations (Figure S5c) show a well-defined and spatially homogeneous electronic structure with a sharp STS peak around the CBE. In this case,

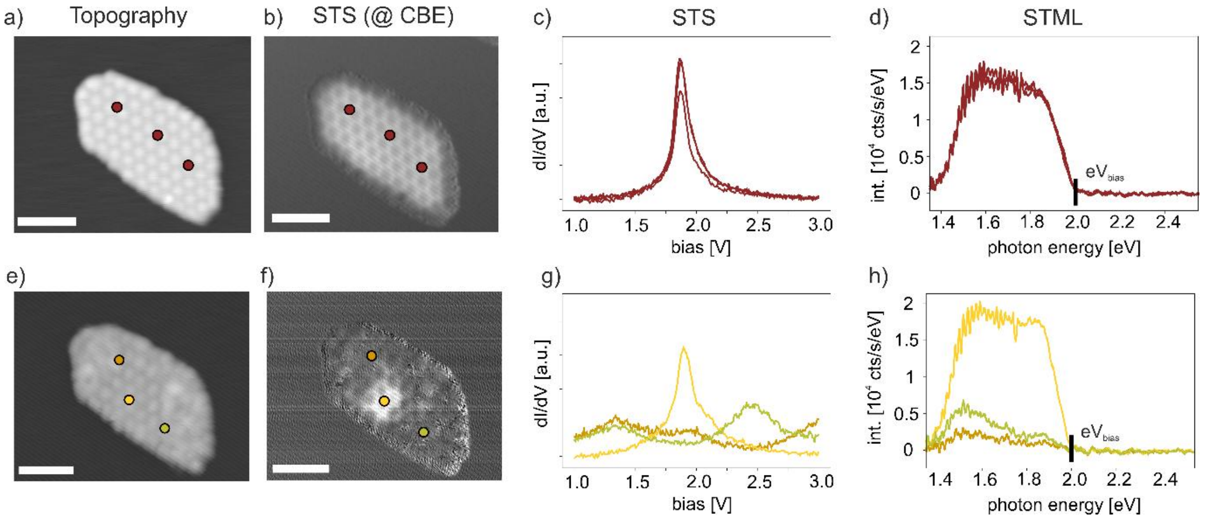

Figure S5. (a-d) STS image, STS map at the CBE, STS scan, and STML spectra of the intact 2ML-ZnO island. (e-h) Same measurements after destruction of the ZnO island induced by tunneling at V_{bias} = 3.7 V and I_t = 8 nA at 78 K. The dots in the STM/STS images indicate the positions of the STS and STML measurements. STM images are measured at V_{bias} = 1 V and I_t = 100 pA. White scale bars are 5 nm. All STS data is measured I_t = 100 pA, STS maps are recorded at V_{bias} = 1.85 V. STML spectra are measured at 2 V and 8 nA and the black bar indicates the quantum cutoff.

we observe constant STML spectra over the whole ZnO flake (Figure S5d). Note that in this data set, the plasmonic luminescence at high photon energies is limited by the quantum cutoff at 2 eV. Tunneling at 3.7 V at 8 nA causes a partial destruction of the well-ordered ZnO structure, and only a small area (yellow position in Figure S4e) shows an intact moiré pattern and a well-defined electronic structure (Figures S4f and S4g). In this well-defined region, the STML spectra are unchanged. However, in the disordered regions with no well-defined conduction band and moiré pattern, the STML intensity is strongly decreased and the spectral shape is slightly altered. This shows that a (locally) well-defined electronic structure is crucial for inducing plasmonic luminescence via charge injection into ZnO/Ag(111).

6. Normalization of STML spectra to the inelastic tunneling rate

To rule out that the observed spectral modifications originate from a modified ineöastic tunneling rate, we normalize the raw STML spectra using the procedure outlined by Martín-Jiménez et al.[3] For this purpose, I-V curves (Fig. S6b) were measured at the same setpoint as that used for the STML spectra (Fig. S6a) on 2ML-ZnO and bare Ag(111). The normalized spectra on ZnO/Ag(111) differs significantly from that on bare Ag(111), as can be seen in Figure S6c). This strongly indicates that the observed low-pass filtering of plasmonic luminescence is not due to an altered inelastic tunneling rate due to a changed final LDOS. In addition, we note that if the plasmonic emission on ZnO/Ag were modified by a different final

state LDOS and a consequently modified inelastic tunneling rate compared to bare Ag, we would expect an energetically shifted cutoff in the bias dependence, as observed e.g. by N. Krane et al.[4]

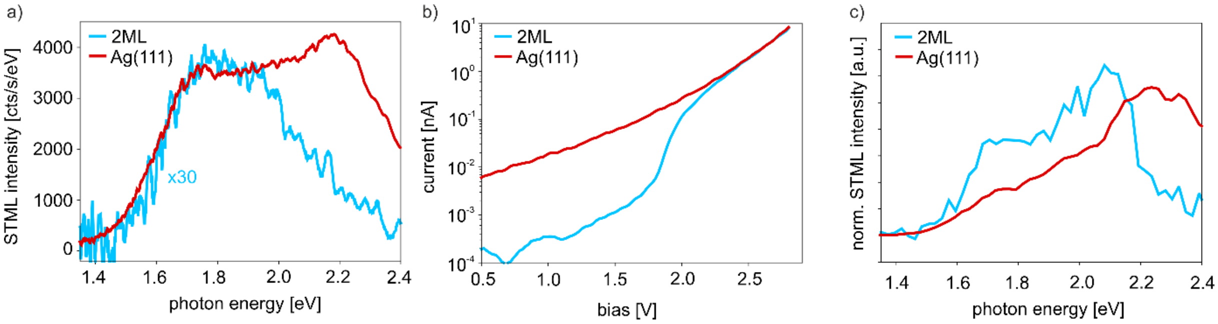

Figure S6 a) STML spectra on 2ML-ZnO (light blue) and Ag(111) (red) at 2.8 V and 8 nA. The spectra on ZnO is rescaled for better comparison. b) Tunneling current as a function of the bias voltage in constant height mode (set point: 2.8 V, 8 nA). c) Normalized STML spectra on 2ML (light blue) and Ag(111) (red) (according to Martín-Jiménez et al.[3]).

References

(1) Kröger, J.; Doppagne, B.; Scheurer, F.; Schull, G. Fano Description of Single-Hydrocarbon Fluorescence Excited by a Scanning Tunneling Microscope. *Nano Lett.* **2018**, *18* (6), 3407–3413. https://doi.org/10.1021/ACS.NANOLETT.8B00304.

(2) Imada, H.; Miwa, K.; Imai-Imada, M.; Kawahara, S.; Kimura, K.; Kim, Y. Single-Molecule Investigation of Energy Dynamics in a Coupled Plasmon-Exciton System. *Phys. Rev. Lett.* **2017**, *119* (1), 013901. https://doi.org/10.1103/PHYSREVLETT.119.013901.

(3) Martín-Jiménez, A.; Fernández-Domínguez, A. I.; Lauwaet, K.; Granados, D.; Miranda, R.; García-Vidal, F. J.; Otero, R. Unveiling the Radiative Local Density of Optical States of a Plasmonic Nanocavity by STM. *Nat. Commun.* **2020**, *11* (1), 1–8. https://doi.org/10.1038/s41467-020-14827-7.

(4) Krane, N.; Lotze, C.; Läger, J. M.; Reecht, G.; Franke, K. J. Electronic Structure and Luminescence of Quasi-Freestanding MoS2 Nanopatches on Au(111). *Nano Lett.* **2016**, *16* (8), 5163–5168. https://doi.org/10.1021/ACS.NANOLETT.6B02101.
